# Trend of measles outbreak in Galmudug, Somalia: a retrospective study from 2023 to 2024

**DOI:** 10.3389/fpubh.2026.1755840

**Published:** 2026-04-17

**Authors:** Abdulkadir M. Ahmed Keynan, Ahmed Mohamed Dirie, Abdiwali Mohamed Ahmed, Shafie Abdirahman Dirie, Abdullahi Abdihakim Ahmed, Abdirahman Mohamed Abdullahi, Osman Abubakar Fiidow

**Affiliations:** 1 Department Research, Training and Disease Surveillance at Banadir Hospital, Mogadishu, Somalia; 2 Faculty of Medicine and Surgery, Benadir University, Mogadishu, Somalia; 3 Faculty of Medicine and Health Sciences, Jamhuriya University of Science and Technology, Mogadishu, Somalia; 4 Department of Community Health, Faculty of Medicine and Health Sciences, University Putra Malaysia, Serdang, Selangor, Malaysia; 5 Department of Health System Strengthening, Ministry of Health, Galmudug, Somalia; 6 Faculty of Medicine and Surgery, Somali National University, Mogadishu, Somalia; 7 Department of Nursing, Faculty of Health Science, Salaam University, Mogadishu, Somalia; 8 Health Emergency Department, Federal Ministry of Health, Mogadishu, Somalia; 9 Department of Public Health, Faculty of Medicine and Health Science, Simad University, Mogadishu, Somalia; 10 Department of Research and Development, Somali Center for Research and Consultancy, Mogadishu, Somalia

**Keywords:** Galmudug, measles, Somalia, surveillance, trend, vaccination

## Abstract

**Background:**

Measles remains a significant public health concern in Somalia, with recurrent outbreaks despite the availability of vaccines. This retrospective study aimed to examine the trends of measles outbreaks in Galmudug State, Somalia, between April 2023 and December 2024.

**Methods:**

A retrospective cross-sectional study was conducted using surveillance data from the Galmudug State Public Health Laboratory. All suspected and laboratory-confirmed measles cases reported between April 2023, and December 2024 were included. Data was analyzed using Microsoft Excel and SPSS version 26, focusing on demographic characteristics, temporal distribution, and IgM confirmation rates. Descriptive statistics, the chi-square (χ^2^) test and logistic regression analyses were performed, with statistical significance set at *p* < 0.05.

**Results:**

A total of 1,705 suspected measles cases were reported during the study period, of which 104 (6.0%) were laboratory confirmed. The incidence rate increased from 4.9 per 10,000 population in 2023 to 8.1 per 10,000 in 2024. The highest burden occurred among children aged 5–9 years (30%), followed by those aged 0–4 years (25.9%). Dusamareb District accounted for 95.8% of reported cases. No significant gender difference was observed (*p* = 0.639).Logistic regression analysis also revealed that the rate of IgM-positive test in children aged 10–14 years and 0–4 years was significantly higher (*p* < 0.05) by 11.3 and 7.7%, respectively, compared to other age groups.

**Conclusion:**

The resurgence of measles in Galmudug indicates persistent immunity gaps and inadequate vaccination coverage, particularly among young children. Strengthening routine immunization, ensuring equitable access to vaccines, and enhancing surveillance and outbreak response mechanisms are critical to achieving measles elimination targets in the region.

## Introduction

Measles is a highly contagious illness caused by the Morbillivirus hominis. Its an enveloped, single-stranded, negative-sense RNA virus (1). The virus primarily spreads through airborne respiratory droplets, and more than 90% of suspected patients may develop symptoms like fever, malaise, cough, runny nose (rhinitis), conjunctivitis, and Koplik's spots, which are typically followed by a maculopapular rash (2, 3).

Before the introduction of the measles vaccine in 1963, the virus caused more than 2 million deaths annually and an estimated 15,000–60,000 cases of blindness worldwide each year (4). Over the past 5 decades, vaccination programs are estimated to have prevented approximately 94 million deaths worldwide (5). Despite a global commitment across all six World Health Organization (WHO) regions to eliminate measles, no region had fully achieved and maintained elimination status by the end of 2023 (6). In response to this ongoing challenge, the Immunization Agenda 2030 (IA2030) identifies measles elimination as a key indicator of immunization program performance. It highlights the importance of robust surveillance systems to detect immunity gaps and calls for achieving equitable 95% coverage with two timely doses of measles-containing vaccine (MCV) (7).

In 2024, an estimated 395,521 laboratory-confirmed measles cases were reported worldwide, reflecting a significant rise from over 300,000 cases in 2023 (7, 8). Sub-Saharan Africa has been particularly affected by this resurgence. As of January 2022, the region reported around 17,500 measles cases a dramatic 400% increase compared to 2021 figures (9). Countries experiencing new outbreaks include Angola, Burundi, Cameroon, the Central African Republic, Chad, the Democratic Republic of Congo, Ethiopia, Somalia, South Sudan, and Togo (10). This outbreak is a consequence of the COVID-19 pandemic, which led to significant immunization gaps across much of the region.

Nationally, Measles remains a major public health concern in Somalia, characterized by recurrent outbreaks and sustained transmission. The largest outbreak in recent years occurred in 2017, when 23,039 suspected cases were reported nationwide. Subsequently, measles transmission persisted, particularly in drought-affected districts, with 2,596 suspected cases reported in 2020, increasing to 7,494 cases in 2021 (11). Epidemiological studies in Somalia have estimated measles case fatality ratios ranging from approximately 2.2%−3.3% in outbreak settings, with higher fatality (up to 11.5%) among displaced children under 5 years of age (12).

Although measles vaccines are sufficiently available in Somalia, outbreaks continue to occur annually. This could be attributed to low vaccination uptake rates. Based on the 2020 Somali Demographic Health Survey (DHS), only 11% of children aged 12–23 months received the measles vaccine (13). Similarly, according to the database from the Somalia District Health Information Software (DHIS2) for the period 2018–2022, the average coverage for the first dose of the measles-containing vaccine (MCV1) was 67.7%, while uptake of the second dose (MCV2) was significantly low by 3.3% (12). This may be due to the recent introduction of MCV2 into the routine childhood immunization schedule in November 2021. Meanwhile, other obstacles also contribute to the persistence of measles in the country. Primarily, long-standing armed conflict, the impact of decades of civil war, recurring droughts, poor access to health services, and limited public health awareness. Therefore, this retrospective study aimed to examine measles outbreak trends in Galmudug State, Somalia, from April 2023 to 2024 to inform evidence-based prevention and preparedness efforts.

## Methods

### Study setting

**Figure 1:**
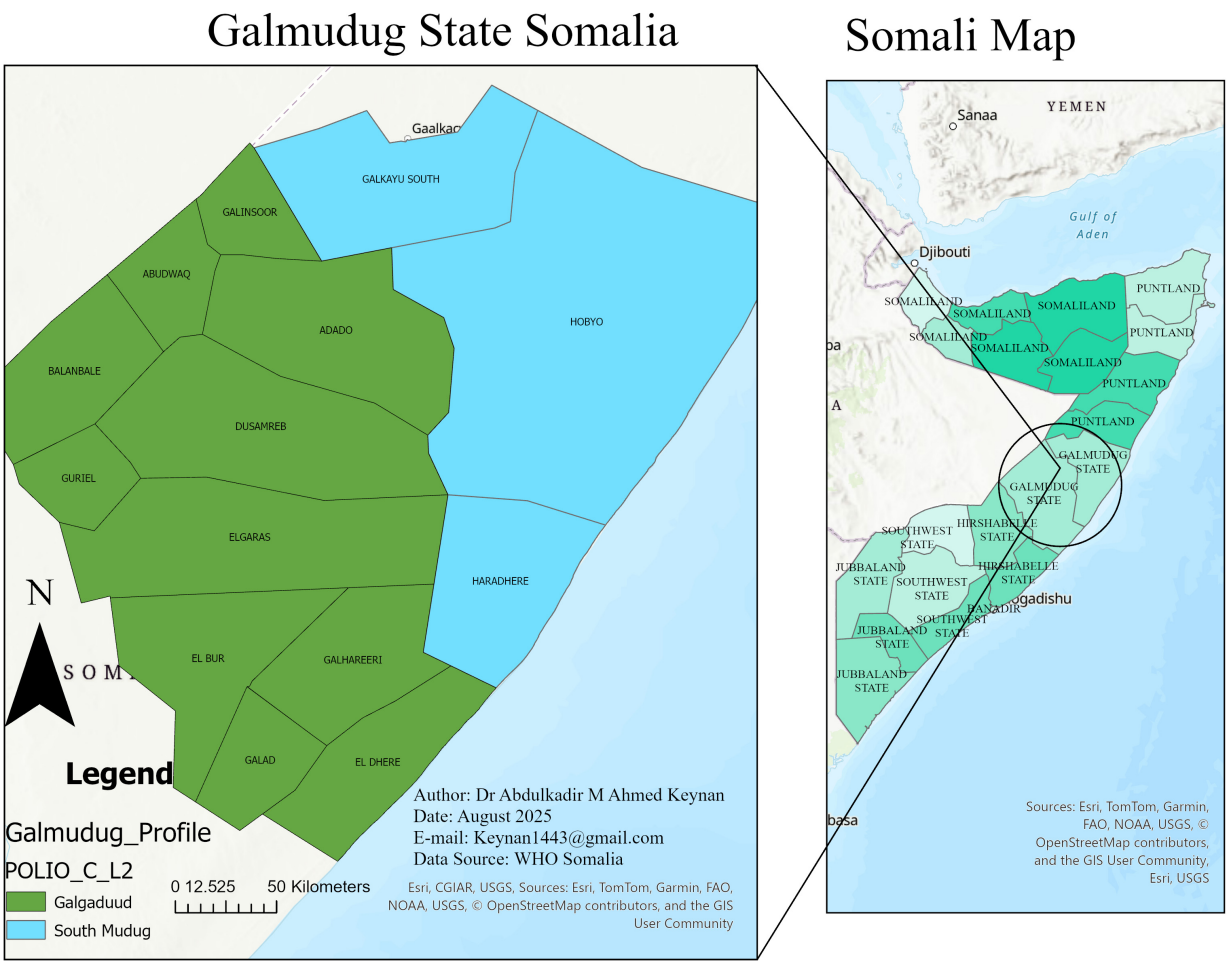
A visualization of the research area and a map displaying the several districts in Somalia's Galmudug state.

Galmudug State was the site of this investigation. Galmudug is located in the central part of Somalia, which is divided into 14 districts (Figure 1). Dusmareb City is the capital of politics and administration. The climate of Galmudug is hot and tropical, with some seasonal variations. According to the latest population estimation survey (mid-2025), Galmudug State is home to approximately 1.4 million people. The population was categorized into urban, rural, and nomadic communities, with the remaining proportion comprising internally displaced persons and other mixed settlement groups (14).

### Study design

A 2-year retrospective cross-sectional study was conducted to analyze the trend of measles outbreaks in Galmudug state, Somalia. The target population covered data recorded in a line list format between April 1, 2023, and December 31, 2024. Data with incomplete documentation (missing data) was excluded from the study.

### Case classification

In this study, the data of measles cases obtained were categorized according to the WHO standard case definitions (15) into the following groups:

1. Laboratory confirmed measles: a person who fulfills the clinical case definition and has laboratory evidence of measles virus infection (measles IgM positive), and no history of measles vaccination in the 30 days prior to specimen collection.
2. Measles confirmed by epidemiological linkage: a person who meets the clinical case definition and has had contact with a laboratory-confirmed measles case.
3. Clinically confirmed/compatible measles: a person who meets the clinical case definition but for whom no laboratory testing has been performed.
4. Discarded/not measles: a suspected case that was fully investigated and either IgM negative or IgM positive due to measles vaccination within 30 days before specimen collection.

### Data collection and analysis

Measles case data, including both laboratory-confirmed and suspected cases suspected cases, were collected by surveillance personnel and systematically entered the electronic database maintained by the State Public Health laboratory, Laboratory confirmation of measles cases was performed by detecting anti-measles IgM antibodies using the enzyme-linked immunosorbent assay (ELISA). Data extracted from the databases included the date of symptom onset (recorded by epidemiological week, month, and year), demographic characteristics (including age, sex, and address), case classification (laboratory-confirmed or suspected cases), vaccination status (unvaccinated, one dose, two doses, or three or more doses), and case outcomes. Cases were stratified into the following age groups: 0–4 years, 5–9 years, 10–14 years, 15–19 years, 20–24 years, and 25 years or older.

The measles incidence rate per 10,000 people was determined by dividing the number of documented measles cases by the total at risk population, with population data sourced from the Population Estimation Survey of Somalia (PESS) database. Additionally, the overall incidence rate was computed, using the population size at risk as the denominator for the incidence rates. Microsoft Excel was utilized to extract data from the measles surveillance database. The collected data were organized as quantitative variables and summarized using frequencies and percentages, which were displayed in tables and figures. Statistical analyses were performed using the Statistical Package for Social Sciences (SPSS), version 26. The chi-square (χ^2^) test was used to assess the association between dependent and independent variables. Additionally, logistic regression was performed to determine the strength of these associations. A *p*-value of < 0.05 was considered statistically significant.

### Result

In this retrospective study, a total of 1,705 suspected measles cases were reported between April 2023 and December 2024. Among these, 104 cases (6.0%) were confirmed (IgM) through laboratory testing. Data analysis also revealed a significant increase in the measles incidence in Galmudug State, rising from 643 cases (4.9 per 10,000 population) in 2023 to 1,062 cases (8.1 per 10,000) in 2024. Based on vaccination status and travel history, none of the suspected cases had received a recent measles vaccination or reported travel history prior to illness In terms of age distribution, the highest burden of measles cases was observed among children aged 5–9 years, accounting for 30.0% of the total cases. This was followed by the 0–4 years age group at 25.9% and those aged 10–14 years at 23.3%. The lowest proportion was reported among individuals aged 25 years and older, representing only 2.8%. The gender distribution remained consistent across both years, with no significant difference observed (*p* = 0.639; Table 1).

**Table 1:** Annual incidence rates and Socio characteristics of reported measles in Galmudug State/Somalia, from April 1, 2023, to December 31, 2024.

Variables		Year			Chi-square value (*P* value)
	**Category**	**2023**	**2024**	**Total (%)**	
Galmudug incidence rate of measles^*^		4.9	8.1		
Total sample size (n%)		643 (37.7)	1,062 (62.3)	1,705 (100)	
Measles vaccination (none, n%)		643 (100)	1,062 (100)	1,705 (100)	
Travel history (none, n%)		643 (100)	1,062 (100)	1,705 (100)	
**Age group (n%)**					148.274, (< 0.001)
	0–4 years	262 (59.3)	180 (40.7)	442 (100)	
	5–9 years	198 (38.7)	314 (61.3)	512 (100)	
	10–14 years	105 (26.4)	292 (73.6)	397 (100)	
	15–19 years	47 (21.2)	176 (78.9)	223 (100)	
	20–24 years	16 (19.3)	67 (80.7)	83 (100)	
	≥25 years	15 (31.3)	33 (68.8)	48 (100)	
**Gender (n %)**					0.220 (0.639)
	Male	317 (37.2)	536 (62.8)	853 (100)	
	Female	326 (38.3)	526 (61.7)	852 (100)	
IgM-result					0.026 (0.871)
	No. of suspected cases	603 (37.7)	998 (62.3)	1,601 (100)	
	No. of confirmed cases	40 (38.5)	64 (61.5)	104 (100)	

^*^Incidence rates per 10,000 population.

**Figure 2:**
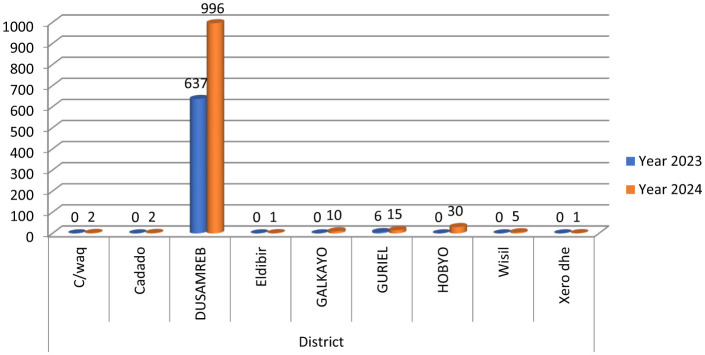
Number of measles cases in Galmudug districts from April 1, 2023, to December 31, 2024 statistically significant association was observed between district and year of reporting (χ^2^ = 18.00, df = 9, *p* = 0.035).

Dusamareb District recorded the overwhelming majority of measles cases, reporting 1,633 cases (95.77%) of the total. In contrast, Hobyo and Guriel districts reported substantially fewer cases, 30 (1.76%) and 21 (1.20%), respectively. A statistically significant association was observed between district and year of reporting (*p* = 0.035), as illustrated in Figure 2.

**Figure 3:**
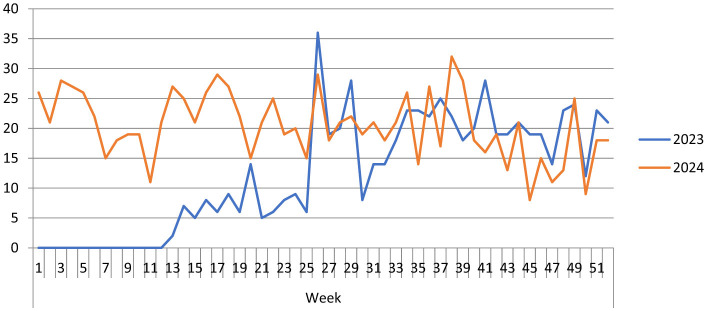
Weekly epidemiological trends of total measles cases in Galmudug state, April 1, 2023, to December 31, 2024.

Figure 3 illustrates that the largest number of suspected measles cases was documented in week 26 of 2023, with a total of 36 cases, followed by week 38 of 2024, which reported 32 cases. In contrast, the lowest number of cases was recorded in week 13 of 2023, with just 2 cases, and week 45 of 2024, which saw 8 cases.

**Figure 4:**
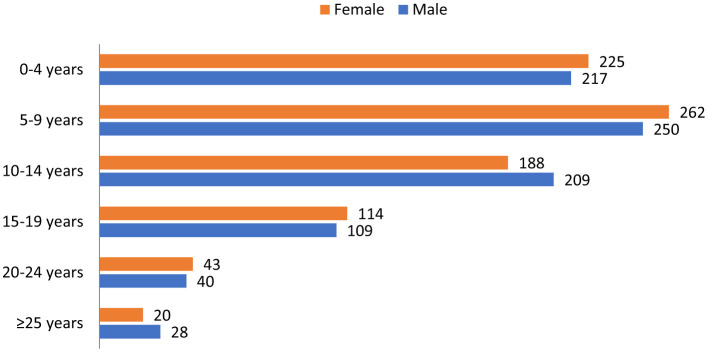
Distribution of measles cases by age group and gender. No association was observed between the age group and gender (χ^2^ = 0.00, df = X, *p* = 1.00).

Figure 4 illustrates the distribution of measles by age group and gender. Females generally showed higher case number than males in the age groups of 0–4 and 5–9 years. However, in the 10–14 and ≥25 years age groups, males had a higher number of cases than females. However, these differences represent descriptive variations only. Statistical testing showed no significant association between age group and gender distribution (*p* = 1.00).

Table 2 presents the multivariate logistic regression analysis of factors associated with IgM results. Significant associations were found between age groups and the likelihood of measles cases. Children aged 5–9 years (AOR = 0.19; 95% CI: 0.09–0.42) were significantly association to the test IgM-positive for measles compared to the reference group of children aged 0–4 years. Similarly, adolescents aged 15–19 years (AOR = 0.45; 95% CI: 0.20–0.98) also was a significantly association of measles IgM positivity than the reference group.

**Table 2:** Multivariate logistic regression analysis of factors associated with IgM results.

Variables	IgM					Reference
	**Category**	**Suspected measles (%)**	**Confirmed measles cases (%)**	**COR (95% CI)**	**AOR (95% CI)** ^*^	***P*** **-value**
Age group (n%)	0–4 years (Ref)	408 (92.3)	34 (7.7)			
	5–9 years	504 (98.4)	8 (1.6)	0.19 (0.09–0.42)^*^	0.19 (0.09–0.42)	**< 0.001** ^*^
	10–14 years	352 (88.7)	45 (11.3)	1.53 (0.962.45)	1.52 (0.95–2.43)	0.078
	15–19 years	215 (96.4)	8 (3.6)	0.45 (0.20–0.98)^*^	0.45 (0.20–0.98)	**0.045** ^*^
	20–24 years	74 (88.2)	9 (10.8)	1.46 (0.67–3.17)	1.46 (0.67–3.18)	0.336
	≥25 years	48 (100)	0 (0.0)	–	–	
Gender (n%)	Male (Ref)	795 (93.2)	58 (6.8)			
	Female	806 (94.6)	46 (5.4)	1.28 (0.86–1.91)	1.26 (0.84–1.89)	0.262

^*^p < 0.05.

## Discussion

Globally, over 300,000 measles cases were reported in 2023 (16), underscoring the continued public health burden of the disease, particularly in low-income and fragile settings. Somalia, a low-income country, continues to experience a high measles burden, with 17,361 cases reported annually (17). This burden is comparable to that observed in other low-income and conflict-affected countries, including Afghanistan (77,210 cases), Djibouti (209 cases), Iran (10,620 cases), Pakistan (17,737 cases), Sudan (3,310 cases), and Syria (6,309 cases) (17). These data highlight persistent immunity gaps and challenges in measles control across low-resource and humanitarian contexts.

Measles remains endemic in Somalia, with new cases reported each year. The trend of measles cases in Galmudug State has increased from 643 cases (37.7%) in 2023 to 1,062 cases (62.3%) in 2024. Statistics also indicated that the measles incidence rate in Galmudug increased from 4.9 cases per 10,000 people in 2023 to 8.1 cases per 10,000 people in 2024. This marked increase in measles cases in Galmudug is attributed to low vaccination rates, as well as the implementation of a new surveillance system and the establishment of public health laboratories in the state in March 2023, which have contributed to the detection of more undiagnosed cases (18).

The increase of measles cases is also reported globally, in which measles cases increased 20% in 2023, compared to 2022 (19). This could be attributed to low vaccination rates. This is consistent with our findings indicated a lack of uptake of the measles vaccine among patients. In this regard, a study conducted in Somalia reported that 98.3% of measles cases occurred among unvaccinated individuals (20). This pattern likely reflects limited access to health services, particularly in hard-to-reach and insecure areas, resulting in persistent immunity gaps. Further, low vaccination rates could be associated with the COVID-19 outbreak, which led to delays or cessation of measles vaccine supplies and a shift in priorities from measles prevention to COVID 19 control (21). Additionally, low vaccination coverage and insecurity in certain districts of Galmudug are also contributing factors (12).

Our study reveals that the Dusmareeb district had more measles cases compared to the other districts. A similar pattern has been observed in the capital city of Jubaland State, Kismayo, where higher measles case numbers were reported relative to surrounding districts (20). The rise in measles cases here may be due to the administrative capital of Galmudug, which hosts the regional referral hospital and several well-staffed health centers. This in turn has prompted people to seek treatment, thus revealing and registering more cases within the city borders. A higher proportion of measles cases was observed in the age groups of 0–4 and 5–9 years. This is consistent with other studies indicating that children aged under 10 years are the most affected, although the prevalence is particularly high among those under five (22). The primary reason is that children under 15 years old are more susceptible to measles due to their developing immune system. Additionally, many children in this age group have not received routine vaccinations against the measles virus, largely due to security issues and lack of awareness.

The finding suggests that sex did not significantly influence susceptibility to measles (*p* = 0.639). This observation aligns with findings from a study in Southeast Ethiopia (19). Likewise, national data from seven high-income countries showed a small male predominance (23). Another study conducted in Ethiopia also corroborated this finding (24). In contrast to our findings, studies conducted in Yemen (8) and other regions of Ethiopia, as well as the European Centre for Disease Prevention and Control (ECDC) reports (25), showed variations in sex distribution.

Overall, the findings of this study provide important insight into the epidemiology of measles in Galmudug State during the study period. The increasing number of reported cases, particularly among children under 10 years of age, reflects persistent immunity gaps and ongoing vulnerability to measles transmission in the region. The geographic concentration of cases in Dusmareeb district also suggests differences in health service access and surveillance capacity across districts, which may influence case detection and reporting. While the expansion of surveillance and laboratory diagnostic capacity has likely improved measles detection, the high proportion of unvaccinated cases highlights continuing challenges in achieving adequate population immunity. These findings contribute to the limited evidence on measles epidemiology in Somalia and provide valuable information for understanding disease patterns in fragile and resource-limited settings.

## Strength and limitation

This study is the first published trend analysis on the measles outbreak in Somalia. surveillance data. However, the reliance on routine retrospective data may introduce underreporting and gaps in vaccination records, which could affect the completeness and accuracy of the findings.

The concentration of healthcare facilities in Dusmareeb district may have led to better case detection, potentially biasing the observed measles distribution. Similarly Underreporting of measles cases is common due to limited access to healthcare, lack of awareness, logistical challenges, population dynamics, and health-seeking behavior. Nevertheless, the measles trends observed in Galmudug can offer valuable insights for the entire country.

## Conclusion

Based on the findings of this study, the high burden of measles cases observed in the state, particularly in the capital, indicates the need for intensified surveillance, rapid outbreak response, and focused immunization campaigns in high-transmission urban areas. The complete absence of measles vaccination among study participants highlights critical gaps in routine immunization services, requiring immediate strengthening of vaccine delivery systems, improved access to measles-containing vaccines, and community mobilization to increase uptake. Furthermore, as the majority of cases occurred among children under 15 years of age, supplementary immunization activities and school- or community-based vaccination strategies should prioritize this age group to close existing immunity gaps and prevent future outbreaks.

## Data Availability

The original contributions presented in the study are included in the article/supplementary material, further inquiries can be directed to the corresponding author.
